# Application of Aspartic Acid Racemization for Age Estimation in a Spanish Sample

**DOI:** 10.3390/biology11060856

**Published:** 2022-06-03

**Authors:** Sara C. Zapico, Douglas H. Ubelaker

**Affiliations:** 1 Department of Chemistry and Environmental Sciences, New Jersey Institute of Technology, Tiernan Hall 365, Newark, NJ 07102, USA; 2 Department of Anthropology, NMNH-MRC 112, Smithsonian Institution, Washington, DC 20560, USA; ubelaked@si.edu

**Keywords:** age-at-death, skeletal remains, forensic anthropology, aspartic acid racemization, Spanish sample

## Abstract

**Simple Summary:**

For the correct identification of human skeletal remains, age is one of the key parameters. However, in adult individuals, this estimation is more difficult as it is not based on growing markers but on degeneration of the skeleton and the teeth. Thus, it can be very variable and less precise than age estimation in children and adolescents. The application of biochemical techniques, with their roots in aging research, could help to improve this estimation. This article presents the application of one of these approaches, aspartic acid racemization, to test its accuracy in a Spanish sample. This is based on the conversion of L-aspartic acid, the regular form of the amino acid in our proteins, into D-aspartic acid, its mirror image. The proportions of D-aspartic acid/L-aspartic acid increase with aging, enabling the determination of age in a more precise way than by applying forensic anthropology methodologies. This paper demonstrates that it was possible to apply this technique in a Spanish sample, obtaining accuracies of ±5 years of actual age. Additional studies should be developed to improve these estimates and to combine this technique with forensic anthropology methods.

**Abstract:**

Correct age-at-death estimation in adult individuals is one of the challenges of forensic investigation. Forensic anthropology macroscopic techniques are non-invasive methods for this purpose. However, several methods need to be applied to accurately estimate age, and the difference between chronological and predictive age may still be around ±10 years. New research trends are focused on the inherent process of aging, which produces changes in tissues and organs at different biochemical levels. One of the oldest and most studied approaches in this field is aspartic acid racemization. The accuracy of this technique in age estimation has been widely demonstrated. However, only a few studies have assessed its accuracy in different populations. The aim of this research was to assess the accuracy of aspartic acid racemization in a Spanish sample and its applicability to forensic cases. Dentin from fifteen third molars from two Spanish populations (ages 19–70 years old) was isolated and D and L forms of aspartic acid were detected through GC/MS, according to a previous published protocol. D/L ratios were calculated and after the application of a regression analysis, a formula for age estimation was developed. The results were similar to previous studies, obtaining an R = 0.91 between racemization ratios and age and a mean absolute error (MAE) between chronological and predictive age of 5 years. These results were ratified by leave-one-out cross-validation, as well as the application of the formula to five teeth of a known age. Despite these promising results, this technique is not exempt from drawbacks; thus, further studies are required to apply this methodology to forensic cases and to combine it with forensic anthropology findings.

## 1. Introduction

One of the main issues in forensic anthropology is estimating the age-at-death of an individual. In adults this is based on physiological degeneration of skeletal and dental structures that seem to have some relationship with age. However, this relationship is influenced by many endogenous and exogenous factors. For this reason, the estimated age may diverge from the chronological age in adult individuals [1]. There are many forensic anthropology approaches to estimating the age-at-death in adults based on different parts of the body, including the pubic symphysis and auricular surface [2] and sternal rib ends [3]. In teeth, age can be estimated by applying the Lamendin technique, which is based on three parameters: periodontal recession, which is caused by the “degeneration of the soft tissue surrounding the tooth from neck to root apex”; root translucency, which does not appear before the age of 20 years, as a consequence of deposition of hydroxyapatite crystals in dentin tubuli; and root height [4,5,6]. Using these parameters, Lamendin created a formula to estimate the age-at-death in a French population. Prince and Ubelaker [5] revised and applied this formula to the Terry Collection to analyze the accuracy of Lamendin′s technique. Their findings indicated that sex and population affinity should be taken into consideration when estimating age-at-death based on these parameters [5,6], creating specific formulas for different subpopulations. Both the Lamendin and Prince-Ubelaker formulas were applied to Spanish population groups, with a better accuracy obtained when using the Prince–Ubelaker formulas [6]. Currently, age-at-death estimation in adult individuals is based on a combination of these approaches with the aim of improving these estimates [7]. However, age-at-death assessment with forensic anthropology means can give an estimation ± 10 years of actual age. 

New research trends are focused on the inherent process of aging, which produces changes in tissues and organs at different biochemical levels [8]. The racemization of aspartic acid is one of these alterations. The amino acids that conform our proteins are in what is called “L-form” due to the stereochemical specificity of the enzymes that utilize only the L-enantiomers. Racemization is a natural process that eventually converts these L-amino acids into its specular forms, D-amino acids, producing a mixture of these two forms of the same amino acid called a “racemic mixture” [9]. Racemization should take place in any metabolically stable protein that is not turned over during the life-time of a long-lived mammal. As a consequence of racemization, the introduction of D-amino acids in the proteins, the conformational structure of these proteins is altered to produce changes in their biological activities or chemical properties [10]. These alterations in proteins can be associated with the aging process, defining racemization as one of the hallmarks of aging [11]. Although racemization occurs in all twenty amino acids, among them, aspartic acid has one of the fastest racemization rates, and for that reason it is the preferred amino acid to study when applying this methodology. In 1975, Helfman and Bada analyzed aspartic acid racemization in tooth enamel from living humans, finding an increase in D/L ratios of aspartic acid with age [9]. Since this study, aspartic acid racemization has been analyzed in different tissues, such as dentin, cementum, the human lens, the white matter of the brain, intervertebral discs, elastin, bone and rib cartilage, showing a positive correlation between aspartic acid racemization and age [1]. These studies agree that dentin is the best tissue to estimate age based on accuracy, simplicity, and time required [12]. Despite it′s widely demonstrated accuracy [13,14,15], this methodology has some drawbacks, such as the destruction of the sample teeth, the necessity of several control teeth [13,16] and the confounding influence of high temperatures [17,18].

Thanks to the accuracy and simplicity of this technique, it has been tested in different populations [19,20,21] towards its application in forensic cases. However, it has not been assessed in Spanish populations. The aim of the present study is to evaluate the efficiency of aspartic acid racemization for age estimation in a Spanish sample.

## 2. Materials and Methods

### 2.1. Materials

Fifteen healthy erupted third molars from a Spanish sample (Asturias in the northwest of Spain and Catalonia in the northeast), age range 19–70 years old, were collected from dental clinics. This was the training set from which the formula was created. Since the aim of this study was to improve age estimation in adult skeletons, third molars were chosen because these are the last teeth to erupt and mineralize, indicating that the person is at least 18 years old. In addition, their position in the mouth, fully protected by environmental issues and the fact that they are, in general, less prone to cavities, make them the ideal sample to work with. Another set of five healthy erupted third molars, age range 28–53 years old, was collected from this Spanish sample as a validation set. The age ranges depended on the available samples provided by the dentists. The Smithsonian Institution′s Institutional Review Board approved all procedures related to experimentation with human subjects. As described below, an exemption was granted for this study because teeth are discarded tissue. Informed consent was not necessary from the patients. All of the samples were obtained anonymously; data collected were limited to sex, age and population affinity. See Table 1 for a detailed description of the samples. Teeth were separated from their alveoli, washed with water, dried and placed in plastic bags.

### 2.2. Methods

#### 2.2.1. Teeth Processing

Teeth processing has been previously described by the authors [22]. Briefly, teeth were cleaned and exposed to UV for 15 min on each side of the tooth. Enamel and cementum were removed using a diamond dental burr. Dentin was exposed. The crown of each tooth was separated from the root using a diamond-cutting disc along the midline. The pulp was removed using a spoon excavator. The isolated dentin was mechanically ground with an agate mortar and pestle and divided into aliquots of 200 mg each.

#### 2.2.2. Aspartic Acid Racemization Analysis

The dentin was subjected to hydrolysis by HCl 6N and chemical derivatization according to the Yamamoto and Ohtani protocol [23]. Samples were analyzed in the ISQ™ LT 7000 Single Quadrupole GC-MS System (Thermo Fisher Scientific) including a chiral column to detect and distinguish D and L forms of aspartic acid and determine their abundance.

#### 2.2.3. Statistical Analysis

Regression formula and correlations between chronological and estimated age were developed using SPSS Statistics 27 (IBM).

## 3. Results

### 3.1. Aspartic Acid Racemization Detection

**Figure 1:**
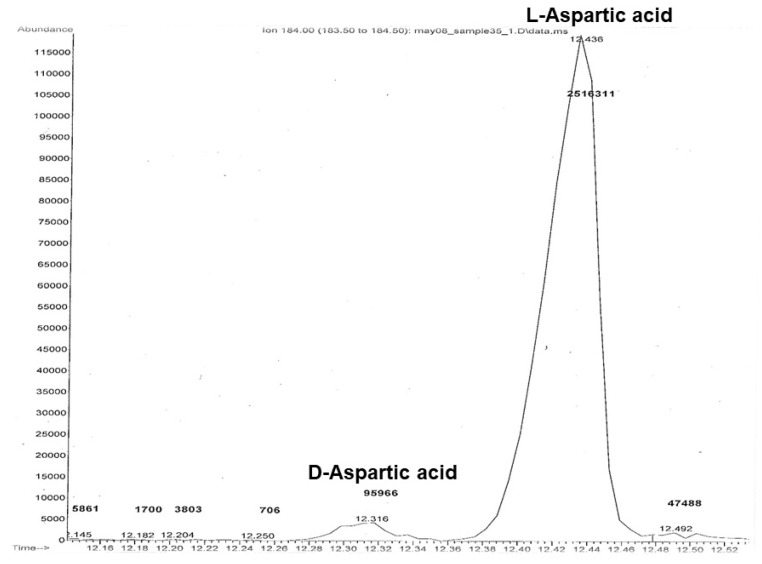
Aspartic acid chromatogram. It shows isomer forms D and L. The *x*-axis represents the retention time; the *y*-axis represents the abundance of the compounds.

Figure 1 shows the mass spectrometry pattern expected to detect D and L forms of aspartic acid. The *x*-axis represents the retention time, when the compounds, in this case L-aspartic acid and D-aspartic acid, were detected by the instrument. The *y*-axis represents the abundance or concentration of L-aspartic acid and D-aspartic acid in the sample, based on standards running on the instrument at the same time as the samples. With age, the D peak will increase, though it will never exceed the L peak. Racemization rates were calculated by converting the integrated peak areas of D-Asp and L-Asp to ln[(1 + D/L)/(1 − D/L)], according to previous protocol [14]. These ratios increased with the age.

### 3.2. Formula for Age-at-Death Estimation Based on Aspartic Acid Racemization Ratios

Using the training set, racemization ratios were calculated based on the formula previously described by Ohtani and Yamamoto (Table 2). These ratios variably increased with age, and moreover in terms of Spanish regions. Based on these ratios, a linear regression formula was created to estimate the age in this Spanish sample:

Age = 2.122 + 619.115 × (ln[(1 + D/L)/(1 − D/L)])

with R = 0.911, R^2^= 0.830, *p* < 0.001, SE = 2.346, mean absolute error (MAE) = 5.6.

Cross-validation of the regression formula was performed by applying leave-one-out as the sample size was small, obtaining an MAE = 4.3.

Additionally, age was estimated based on this formula in the validation set, obtaining accuracies between ±2–6 years of actual age. The results of this validation are shown in Table 3.

### 3.3. Correlation between Chronological and Predictive Ages in the Training Set

**Figure 2:**
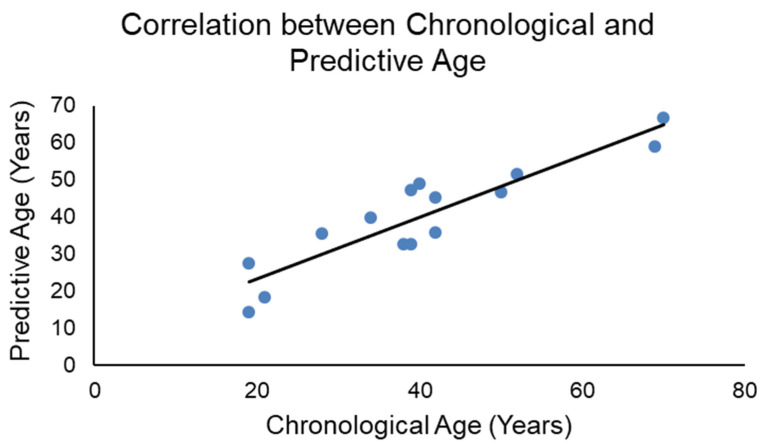
Pearson correlation comparing chronological and predictive age in the training set.

Pearson correlations were performed to compare the predictive ages and chronological ages in the training set (Figure 2), obtaining an R = 0.910 with *p* < 0.001.

## 4. Discussion

Identification of an individual depends on the correct estimation of its biological profile: sex, population affinity, height and age. Age estimation in the adult skeleton is altered by different variables. Due to this, it is important to use tissues and techniques that are not affected by these factors.

The present paper assessed one of the most common and oldest techniques for age estimation by biochemical means: aspartic acid racemization [9]. Since the first and successful attempt in 1975, several works have studied this technique in different samples, evaluating both the best tissue [17,24,25,26,27,28] and methodological approach [23]. These studies agreed that teeth, particularly dentin, and GC/MS are the best choices when applying this technique to forensic cases [13].

Thanks to their structure, teeth can remain in the skeleton after everything else has decomposed. Thus, it is feasible to encounter teeth in skeletonized human remains and apply this technique to complement and improve age estimates in adult individuals.

Recent publications in forensic anthropology have demonstrated that, when developing a new technique for determination of biological profiles, the population of origin should be considered. Some of the most recent studies in aspartic acid racemization precisely assess the application of this technique with different populations [19,21,29,30,31,32]. The present study complements this previous work, being the first time that aspartic acid racemization has been assessed and applied for age estimation in a Spanish sample. Our work agrees with these aforementioned population studies verifying that aspartic acid racemization and, as a result, the racemization ratios increase with age. Although our study and the previous studies used dentin as the chosen tissue, there were differences in terms of the accuracy of the methodology. These differences could be due to the type of tooth used for the analyses [33]. The study by Wochna et al. [21] demonstrated that the use of central incisors provides better age estimates than first premolars, probably due to an increase in temperature in the molar region compared to the front region, as explained by Ohtani et al. [33]. Our study did not compare different teeth. However, the chosen teeth were third molars because of their position in the mouth, fully protected from environmental insults, and because these are the last teeth to erupt and mineralize, functioning as a mark of an adult skeleton [34]. This could be a factor to consider for our lower accuracy compared to other studies, although our results are still in the range expected for the application of this methodology, including the age estimates in the validation set [19,21,29,30,32].

Another important point to consider is the number of teeth used for this study. Fifteen teeth is a low number; however, the study by Wochna et al. [21] assessed between 14 to 21 teeth, depending on the type of tooth, and still obtained good accuracy, particularly for the central incisors. In addition, practical application of this technique to forensic cases requires several control teeth of the same type as the those recovered from the remains and with known ages to assess the racemization ratios and create the regression formula [14,35]. As a result, the number of control teeth could be also low, around ten teeth, depending on the availability.

Additional questions arise related to which part of the tooth should be used. According to the study by Ohtani [28], “whole dentin in longitudinal sections of the central area of the tooth should be examined for accurate estimation of age”. In the present study, we did not distinguish among different dentin parts because pulverized dentin was divided into aliquots for additional DNA analyses. However, specifically selecting and separating dentin from this part of the tooth constitutes a methodological constraint. In fact, Sakuma et al. [36] evaluated the accuracy of this technique when using the whole tooth or only dentin in order to simplify the methodological approach. The correlation between racemization ratios and age in the whole tooth was still high (0.93), but not as high as dentin (0.98). Although this could make this technique easier and could increase the applicability of this technique for age estimation, it should still be considered that the whole tooth is a mixture of different layers with different structures. Our study only focused on dentin because previous work from our group applying other biochemical techniques for age estimation [37,38,39] highlighted the importance of tissue-specificity in the accuracy of the technique.

It is important to mention the source of the teeth in our study. Although they came from a Spanish population, they constituted a mixture of two Spanish populations. Ten teeth came from Asturias, in the northwest of Spain, and five teeth came from Catalonia, in the northeast. Racemization ratios seemed to differ for the same age between these two populations, probably due to different environmental conditions. Previous studies by our group demonstrated these differences [37]. However, in the present work, we combined these two populations because, when the aspartic acid racemization technique is applied for a forensic case, several control teeth are needed to develop the formula for age estimation. Nevertheless, we are aware that this may be a factor that decreases the accuracy of the technique. Further studies are needed to confirm this population-specificity.

In this study, we did not assess different situations that could affect the estimates based on this technique. However, previous studies have pointed out that fire, acidic burials and other environmental conditions can hamper the accuracy of this estimate [40,41,42,43].

With respect to the utility of this technique in forensic cases, it is principally Ohtani and Yamamoto who have demonstrated its applicability [13,14,35,44]. The instrumentation required (GC/MS) is available in any forensic lab, as it is also used for drug/toxicological analyses. Furthermore, the procedure and the derivatization and processing of the samples can be carried out by any forensic chemist/biochemist or individual with basic knowledge of chemical sample preparation. The only critical, and the most difficult, step is the separation of the tooth layers and the isolation of the dentin, which can be time-consuming. Thus, regarding the applicability of this technique to forensic cases, forensic scientists should assess the benefits of its performance, such as increasing the accuracy of age estimates, vs. other factors, such as the time requirements.

## 5. Conclusions

This work demonstrated for the first time the applicability of the aspartic acid racemization technique for age estimation in a Spanish sample. Though the accuracy was not as high as previous studies, it was still higher than age estimates using classical forensic anthropology techniques. Further studies should complement these results by increasing the number of teeth samples and assessing this technique in other Spanish regions. In addition, future work should aim to collect teeth samples from corpses in order to analyze the impact of different factors on this technique, such as the postmortem interval and other environmental insults.

## Figures and Tables

**Table 1:** Description of teeth samples used in this study including age, sex, population affinity and Spanish region.

	**Patient**	**Age**	**Sex**	**Population Affinity**	**Spanish Region**
**Training Set**	1	19	Male	Caucasian	Asturias
	2	19	Female	Caucasian	Catalonia
	3	21	Male	Caucasian	Catalonia
	4	28	Male	Caucasian	Asturias
	5	34	Male	Caucasian	Asturias
	6	38	Male	Caucasian	Catalonia
	7	39	Male	Caucasian	Asturias
	8	39	Male	Caucasian	Catalonia
	9	40	Male	Caucasian	Asturias
	10	42	Female	Caucasian	Asturias
	11	42	Male	Caucasian	Catalonia
	12	50	Female	Caucasian	Asturias
	13	52	Female	Caucasian	Asturias
	14	69	Male	Caucasian	Asturias
	15	70	Male	Caucasian	Asturias
**Validation Set**	1	28	Female	Caucasian	Asturias
	2	34	Male	Caucasian	Asturias
	3	43	Female	Caucasian	Asturias
	4	52	Female	Caucasian	Asturias
	5	53	Female	Caucasian	Asturias

**Table 2:** Aspartic acid racemization rates with respect to the chronological age in a Spanish sample; training set. Age was estimated based on these racemization rates and the regression formula. In black, teeth from Asturias population; in red, teeth from Catalonia population.

Patient	Age	ln (1 + D/L)/(1 − D/L)	Estimated Age
1	19	0.040987189	27
2	19	0.019799151	14
3	21	0.026196231	18
4	28	0.053827959	35
5	34	0.061061632	39
6	38	0.049286225	33
7	39	0.049269116	33
8	39	0.072825693	47
9	40	0.075748087	48
10	42	0.05437045	37
11	42	0.069579025	45
12	50	0.071876747	47
13	52	0.07994421	51
14	69	0.091899884	59
15	70	0.10427175	67

**Table 3:** Aspartic acid racemization rates with respect to the chronological age in the validation set. Age was estimated based on these racemization rates and the regression formula.

Patient	Age	ln (1 + D/L)/(1 − D/L)	Estimated Age
1	28	0.052934746	34
2	34	0.054791161	36
3	43	0.060826149	40
4	52	0.072133607	47
5	53	0.086259913	55

## Data Availability

The data presented in this study are available on request from the corresponding author. The data are not publicly available due to privacy issues.
